# Investigating the influence of thought interference and somatic passivity on outcomes in patients with psychosis

**DOI:** 10.1192/j.eurpsy.2021.2132

**Published:** 2021-08-13

**Authors:** T. Magrangeas, A. Kolliakou, J. Sanyal, R. Patel, R. Stewart

**Affiliations:** 1 Institute Of Psychiatry, Psychology And Neuroscience, King’s College London, London, United Kingdom; 2 Biomedical Research Centre, South London and Maudsley NHS Foundation Trust, London, United Kingdom

**Keywords:** Natural-Language Processing, prognosis, schizophrénia, psychosis

## Abstract

**Introduction:**

Of the many studies describing psychotic symptoms in schizophrenia, few have investigated their direct influence on prognosis.

**Objectives:**

We aimed to apply natural language-processing (NLP) algorithms in routine healthcare records to identify reported somatic passivity and thought interference symptoms (thought broadcasting, insertion and withdrawal), and determine associations with prognosis by an analysis of routine outcomes.

**Methods:**

Four algorithms were thus developed on de-identified mental healthcare data from a large south London provider and were applied to ascertain recorded symptoms over the three months following first presentation to that service in a cohort of patients with a primary schizophreniform disorder (ICD-10 F20-F29) diagnosis. The primary binary dependent variable for logistic regression analyses was any negative outcome (Mental Health Act section, >2 antipsychotics prescribed, >22 days spent in crisis care) over the subsequent 2 years, adjusted for age, gender, ethnic group, neighbourhood deprivation, diagnostic group, and recorded paranoia, persecutory delusions or auditory hallucinations.

**Results:**

In 9,323 patients, final models indicated significant associations of this composite outcome with baseline somatic passivity (prevalence 4.9%; adjusted odds ratio 1.61, 95% CI 1.37-1.88), thought insertion (10.7%; 1.24, 1.15-1.55) and thought withdrawal (4.9%; 1.36, 1.10-1.69), but not independently with thought broadcast (10.3%; 1.05, 0.91-1.22).

**Conclusions:**

Symptoms traditionally central to the diagnosis of schizophrenia, but under-represented in current diagnostic frameworks, were thus identified as important predictors of short- to medium-term prognosis.

**Disclosure:**

No conflict of interest - past support from Janssen; GSK; Takeda; Induction Healthcare; Holmusk; the NIHR; SLaM NHS Trust; the MRC; KCL; the NIHR ARC; KCH NHS Trust; the Academy of Medical Sciences; The Wellcome Trust; BHF; Arthritis Research UK; the Roya

